# Dynamics of neuronal oscillations underlying nociceptive response in the mouse primary somatosensory cortex

**DOI:** 10.1038/s41598-021-81067-0

**Published:** 2021-01-18

**Authors:** Shosuke Iwamoto, Makoto Tamura, Atsushi Sasaki, Masao Nawano

**Affiliations:** 1 Neuroscience Research Unit, Mitsubishi Tanabe Pharma Corporation, 1000, Kamoshida-cho, Aoba-ku, Yokohama, Kanagawa 227-0033 Japan; 2 Present Address: NeuroDiscovery Laboratory, Mitsubishi Tanabe Pharma Holdings America, Cambridge, MA 02139 USA

**Keywords:** Neuroscience, Diseases of the nervous system, Chronic pain

## Abstract

Pain is caused by tissue injury, inflammatory disease, pathogen invasion, or neuropathy. The perception of pain is attributed to the neuronal activity in the brain. However, the dynamics of neuronal activity underlying pain perception are not fully known. Herein, we examined theta-oscillation dynamics of local field potentials in the primary somatosensory cortex of a mouse model of formalin-induced pain, which usually shows a bimodal behavioral response interposed between pain-free periods. We found that formalin injection exerted a reversible shift in the theta-peak frequency toward a slower frequency. This shift was observed during nociceptive phases but not during the pain-free period and was inversely correlated with instantaneous pain intensity. Furthermore, instantaneous oscillatory analysis indicated that the probability of slow theta oscillations increased during nociceptive phases with an association of augmented slow theta power. Finally, cross-frequency coupling between theta and gamma oscillations indicated that the coupling peak frequency of theta oscillations was also shifted toward slower oscillations without affecting coupling strength or gamma power. Together, these results suggest that the dynamic changes in theta oscillations in the mouse primary somatosensory cortex represent the ongoing status of pain sensation.

## Introduction

Pain, one of the most unpleasant sensations, is caused by tissue injury, inflammatory disease, pathogen invasion, and neuropathy. The pain signal is mediated by nociceptors on the Aδ- and C-fibers that innervate the skin, joints, and body organs, and is conducted via the spinal cord to the brain, where it is perceived as pain^1^. The perception of pain is attributed to the neuronal activity in the brain. However, the dynamics of the neuronal activity underlying pain perception are not fully known.

Brain rhythmic oscillatory activity is altered in association with pain perception in humans and model organisms^2,3^. Specifically, alpha or theta-band frequency activity in the primary somatosensory (S1) cortex has been linked to pain states in humans and animal models^4^, such that alpha/theta-wave activity in the S1 cortex is augmented in patients with chronic pain^5–7^ and animal models of chronic pain^8^. In addition, the alpha/theta-peak frequency is shifted toward slower frequencies by a nociceptive stimulus in humans^5,6,9,10^, as well as in a mouse model of trigeminal neuropathic pain^11^. However, the dynamic relationship between theta-range oscillation and pain sensation remains unknown. Importantly, it remains unclear whether theta oscillation changes are dynamically associated with time-dependent changes in pain sensation, and whether pain-relevant changes in theta oscillations influence oscillatory activities in the higher frequency range according to theta-oscillation dynamics.

In this study, we examined theta-oscillation dynamics of local field potentials in the S1 cortex of a formalin-induced mouse model of pain, which shows a bimodal pain response interposing a pain-free period. The findings presented here demonstrate that formalin injection exerts a reversible shift of the theta-peak frequency toward slower frequencies. This shift was observed during nociceptive phases but not during the pain-free period and inversely correlated with the instantaneous pain intensity. Furthermore, instantaneous oscillatory analysis indicates that the probability of slow theta oscillations increases during nociceptive phases with the association of augmented slow theta power. Finally, cross-frequency coupling between theta and gamma oscillation indicates that the coupling peak frequency of theta oscillations is also shifted toward slower frequencies without affecting coupling strength or gamma power. Together these results suggest that the dynamic changes in theta oscillations in the mouse S1 cortex represent the ongoing status of pain sensation.

## Results

### Theta-peak shift in the somatosensory cortex linked to nociceptive responses

**Figure 1:**
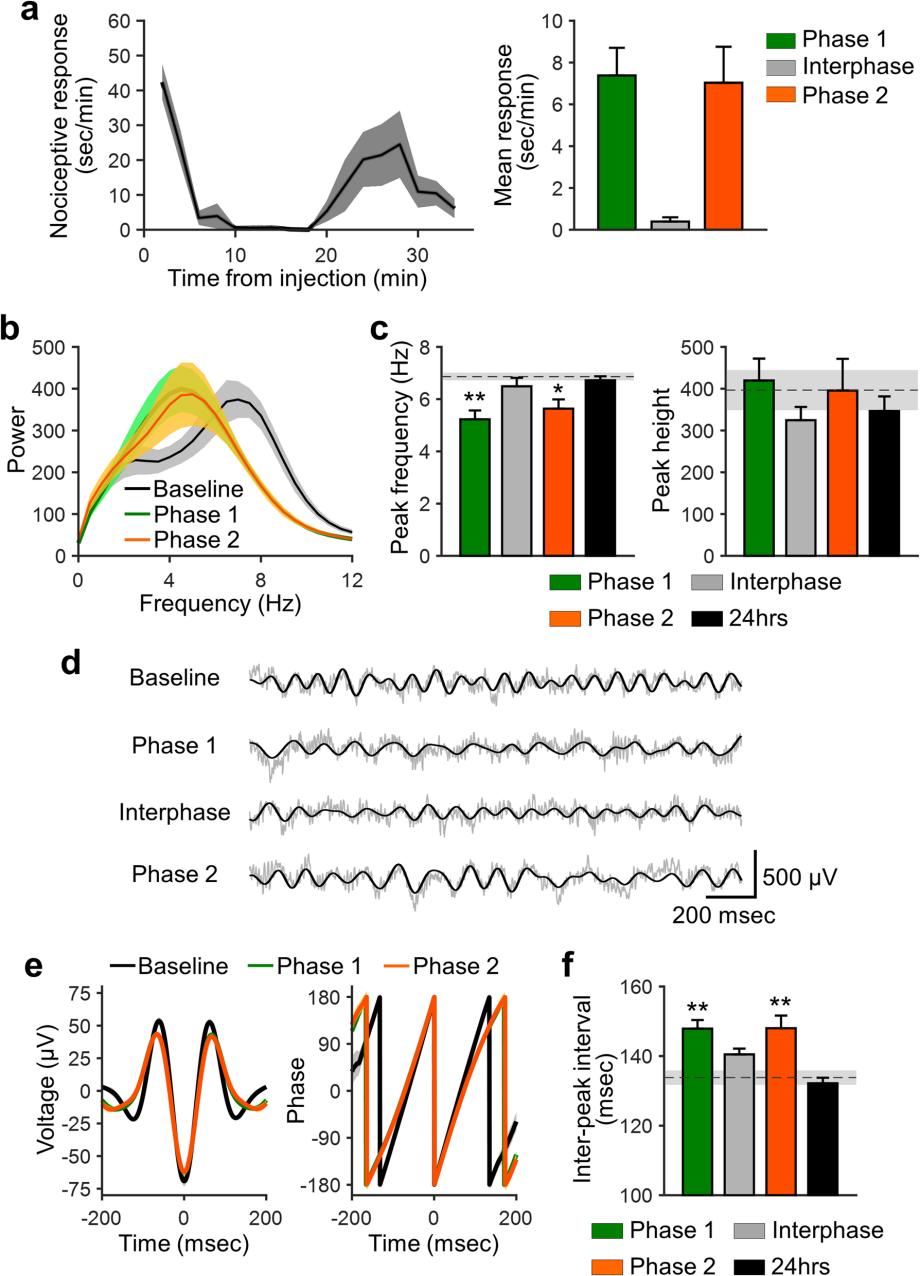
The peak of theta-range oscillations in the somatosensory cortex is shifted toward slow frequency during nociceptive phases. (**a**) Left: nociceptive response (i.e., licking behavior) after injection of 5% formalin into the left hind paw. Right: mean nociceptive response in phase 1 (0–10 min), interphase (10–20 min), and phase 2 (20–35 min). (**b**) Power spectra in the somatosensory cortex at baseline (i.e., before formalin injection) and during nociceptive phases. (**c**) Mean theta-peak frequency (left) and height (right) after formalin injection. The dashed line and shaded area indicate the mean values and the standard error of the mean (SEM) at baseline, respectively. The peak frequency was significantly lower during phases 1 and 2 compared to baseline, but not during the interphase or at 24-h post-injection. The noxious stimulus did not affect the theta-peak height. (**d**) Representative theta oscillations (black) and raw local field potentials (LFPs, gray) before and after injection of formalin. (**e**) Averaged theta-range filtered traces (left) and phases (right) obtained by aligning the signals at the trough of theta oscillations. (**f**) Inter-peak interval of theta-range filtered LFPs. The dashed line and shaded area indicate the baseline mean value and SEM, respectively. The inter-peak interval was significantly longer during phases 1 and 2 than at baseline, but not during interphase or 24-h post-injection; n = 7 animals. Data are presented as mean ± SEM; **P < 0.01; *P < 0.05 vs. baseline values; a repeated measures ANOVA with a post hoc Dunnett’s multiple comparison test.

To investigate the dynamics of pain-relevant oscillatory changes in the S1 cortex, mice were chronically implanted with a microelectrode in the S1 region (Supplementary Figure S1 online). Neural signals were recorded before and after intraplantar injection of 5% formalin into the left hind paw. Peripheral formalin injection is thought to be a model for acute inflammatory pain. Intraplantar injection of formalin generated an initial phase of nociceptive response (phase 1, 0–10 min), a quiescent interphase (10–20 min), and a second phase of nociceptive response (phase 2, 20–35 min; Fig. 1a), as previously described^12–14^. The initial acute response during phase 1 is thought to reflect direct activation of nociceptors in Aδ- and C-fiber afferents, while a prolonged response during phase 2 is probably due to the ongoing inflammatory input and central sensitization, resulting from the barrage of input from Aδ- and C-fiber afferents during the early phase^15,16^.

We first examined the dynamics of local field potentials (LFPs) linked to pain-related behavior. Spectral analysis of LFPs revealed that formalin injection induced a peak shift in theta-range oscillations (4–12 Hz) toward a slower frequency (Fig. 1b). Indeed, the peak frequency of theta waves was significantly decreased during phases 1 and 2 (Fig. 1c). This tendency was not observed during the interphase or at 24-h post-injection. By contrast, the theta-peak height did not differ significantly following formalin injection, regardless of the behavioral phase (phase 1, interphase, phase 2, 24-h post-injection; Fig. 1c). To further examine the relationship between theta-range oscillations and nociceptive response, LFP signals were filtered at the theta-frequency range with Hilbert transform (Fig. 1d). Theta-peak aligned waves and phases are shown in Fig. 1e. This analysis indicated that the inter-peak interval of theta-filtered oscillations was significantly longer during phase 1 and phase 2 than at baseline but not during interphase or 24-h post-injection (Fig. 1f). These data indicate that the theta-range oscillations are slowed down by the noxious stimulus and reversibly changed according to the nociceptive behavioral response.

**Figure 2:**
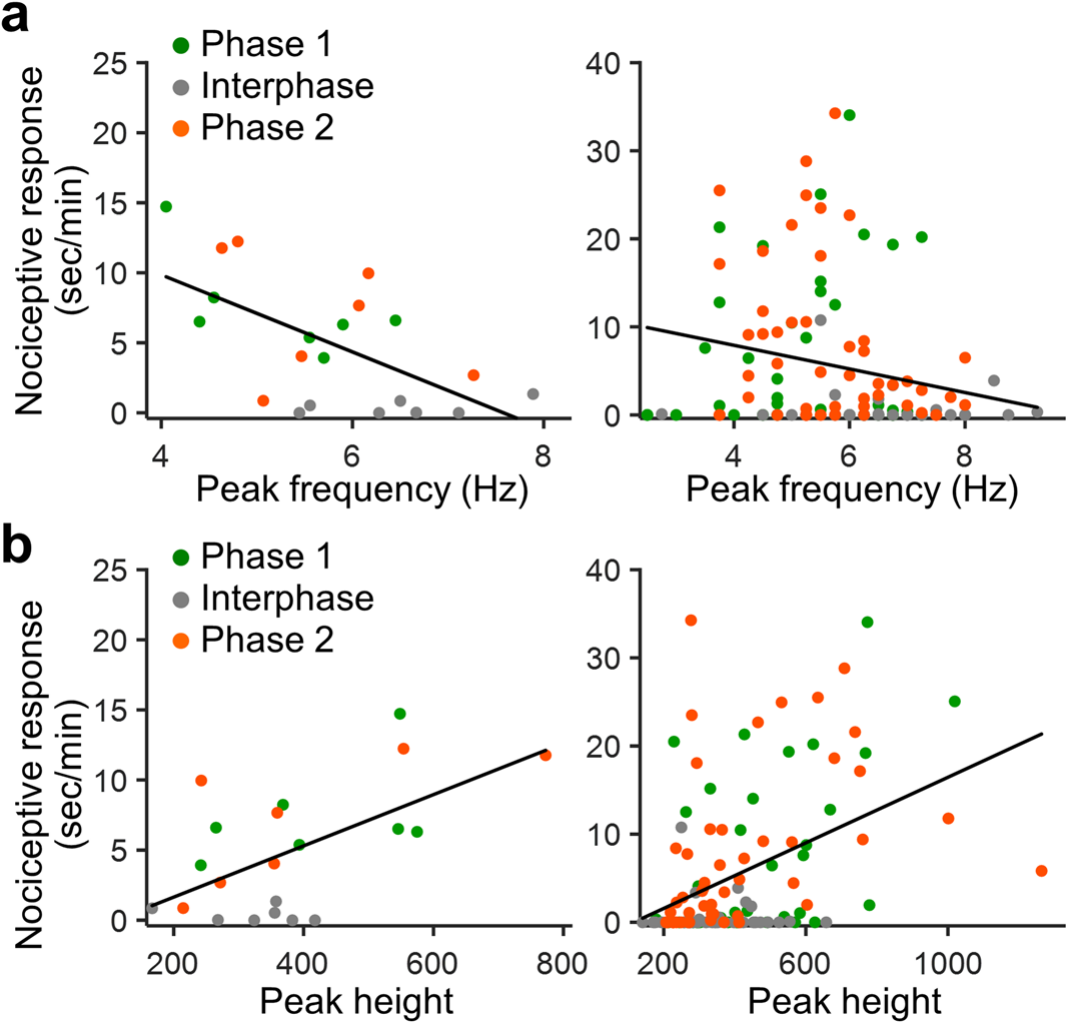
Nociceptive response negatively correlates with the theta-peak frequency and positively with the height. (**a**) Scatter plots depicting nociceptive response plotted against theta-peak frequency. Left: mean values during individual phases in individual animals. Right: values binned by time (2 min) for each mouse. There was a significant inverse correlation of nociceptive response with peak frequency (individual phases: R = − 0.61, P = 0.0036, time-binned values; R = − 0.21, P = 0.018). (**b**) Nociceptive response plotted against theta-peak height. Nociceptive response positively correlates with theta-peak height (individual phases: R = 0.59, P = 0.0049, time-binned values; R = 0.42, P = 0.0000018). R: correlation coefficient, Pearson’s correlation.

Careful examination of the relationship between theta-range oscillations and pain-related behavior indicated that the slower peak of theta-range oscillations was associated with a greater nociceptive response (Fig. 2a). This was applicable for both values on a phase-by-phase basis and time-binned values. Although the theta-peak height did not significantly differ following formalin injection (Fig. 1c), it positively correlated with the nociceptive response, such that the higher theta peak was associated with a greater nociceptive response (Fig. 2b). These data suggest that the peak frequency and the height of theta waves covaried with pain intensity.

### Theta-peak shift associated with peak height in a pain state

**Figure 3:**
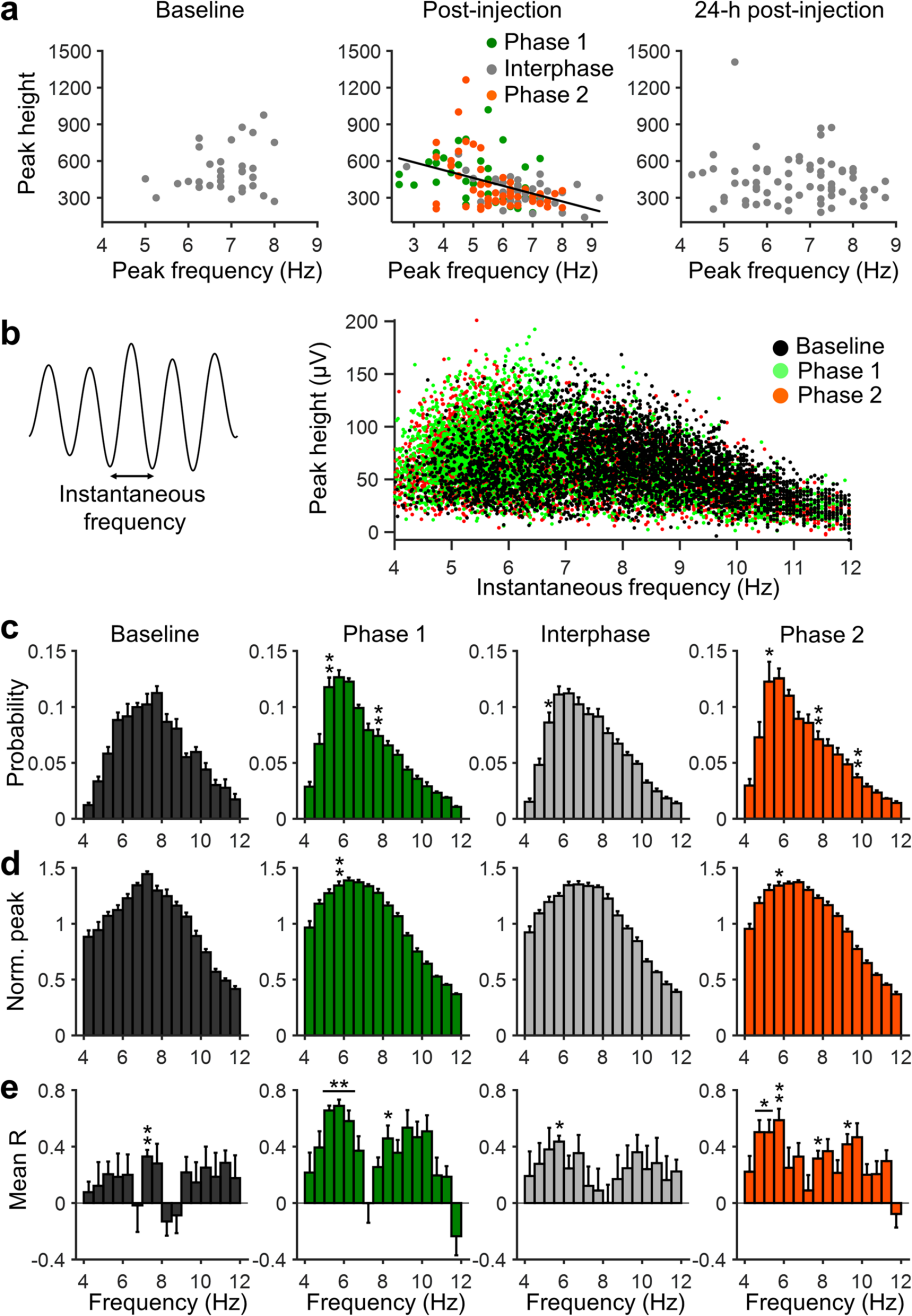
Slow theta oscillations are associated with the theta-peak height during nociceptive responses. (**a**) Scatter plots depicting theta-peak height plotted against theta-peak frequency binned by time (2 min) during pre- and post-injection, and 24-h post-injection. Theta-peak frequency shows significant inverse correlation with the theta peak height at post-injection, but not at pre-injection or 24-h post-injection (baseline: R = 0.21, P = 0.25; post-injection: R = − 0.45, P = 0.00000021; 24-h post injection: R = − 0.084, P = 0.49). (**b**) Left: instantaneous frequency analysis of theta-range-filtered waves. Right: peak frequency plotted against the height of instantaneous theta waves in an example animal. (**c**) Probability of instantaneous theta frequencies. The probability of theta waves is shifted toward the slow frequency after formalin injection, such that the probability is significantly higher at 5 Hz during phase 1, interphase, and phase 2 compared to baseline, and significantly lower at 7.5 Hz during phases 1 and 2. (**d**) Normalized peak height as a function of instantaneous frequency. Peak height is significantly higher at 5.5 Hz during phases 1 and 2 than at baseline. (**e**) Mean correlation coefficient of the probability and peak height of instantaneous theta waves as a function of frequency. There is a significant positive correlation (i.e., significant difference from r = 0) between the probability and peak height at low theta frequency (5–6.5 Hz) during phases 1 and 2. This tendency is not observed at baseline. Data are presented as mean ± SEM; **P < 0.01; *P < 0.05 vs. baseline values (**c**, **d**) or vs. r = 0 (**e**); Student’s *t*-test with Bonferroni correction.

Since both theta-peak frequency and height were significantly associated with nociceptive response, we examined the relationship between theta-peak frequency and height. Correlational analysis revealed that there was a significant inverse correlation between theta-peak frequency and height after formalin injection (Fig. 3a). In other words, the slower the peak frequency, the higher the theta peak. This tendency was not observed in the pre-injection period and at 24-h post-injection, suggesting that a noxious stimulus generated a dynamic link between the theta-peak frequency and height.

We further investigated this relationship by analyzing instantaneous theta oscillations (Fig. 3b). Figure 3c depicts the distribution of instantaneous frequency of theta waves. At baseline, the instantaneous frequency showed a unimodal distribution centered around 7.5 Hz. During nociceptive phases, however, the distribution was left-shifted, and 5-Hz oscillations were most frequently observed (Fig. 3c). Indeed, compared to baseline oscillations, the probability of 5–5.5 Hz oscillation frequencies was significantly increased, whereas that of 7–7.5 Hz oscillations was significantly decreased during nociceptive phases (Fig. 3c). Furthermore, the distribution of the normalized peak height at each frequency was altered by the noxious stimulus, such that the height of slower oscillations (5.5–6 Hz) was significantly higher during nociceptive phases than at baseline (Fig. 3d).

These data indicate that an increase in probability of slow theta waves is associated with an increase in the height of oscillations. Indeed, a significant positive correlation was observed around slower frequencies (i.e., 4.5–6 Hz) between probability and height during nociceptive phases (Fig. 3e); the higher the probability of slower theta waves, the higher the peak of oscillations. This was not the case at baseline, such that significant positive correlation was observed only at 7–7.5 Hz oscillations. This indicates that increased probability and peak height in slower theta frequencies coordinately represent a nociceptive response.

### Distinct associations of subdivided theta waves with nociceptive response

**Figure 4:**
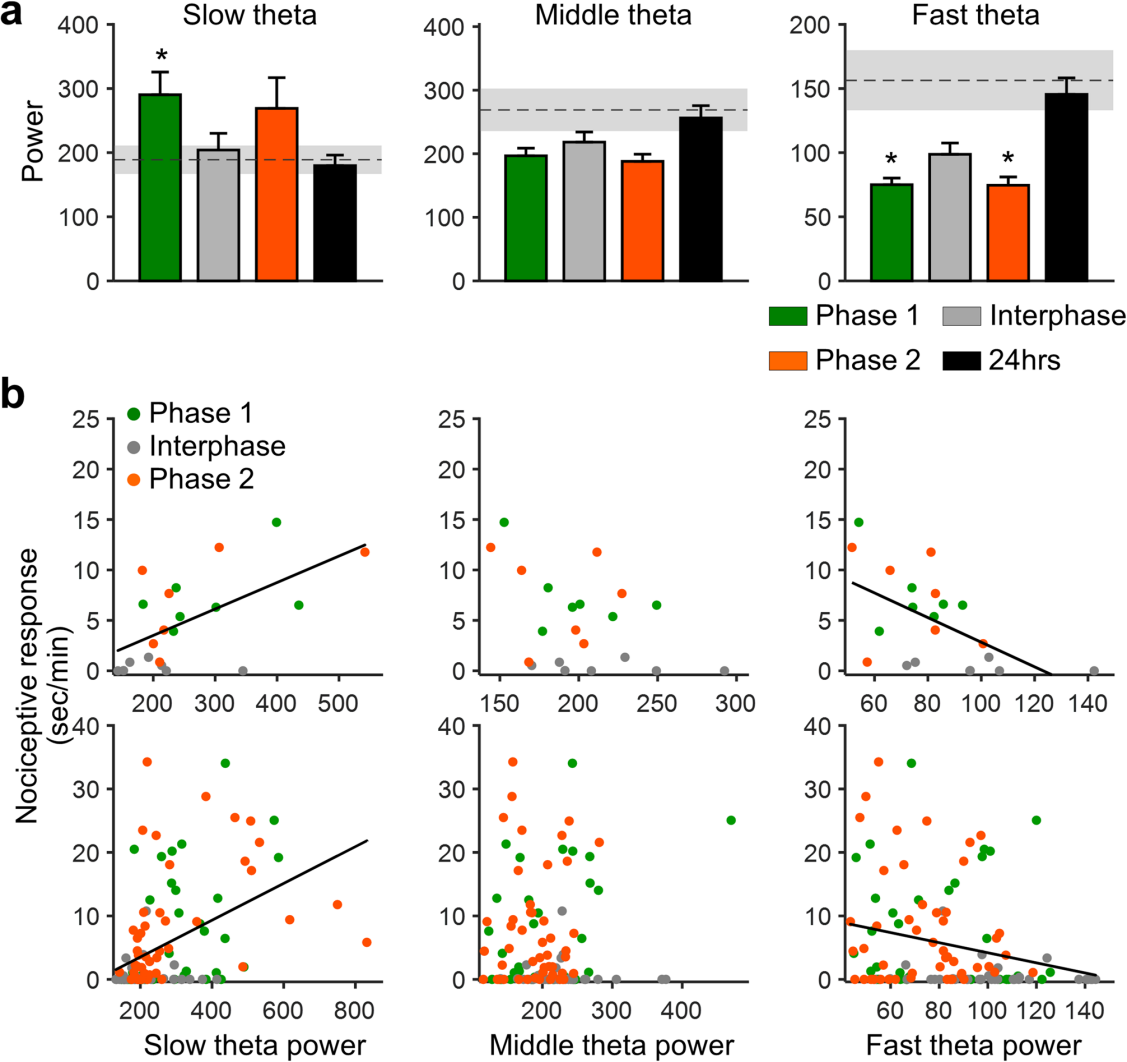
Nociceptive response positively and negatively correlates with slow and fast theta power, respectively. (**a**) Mean power in slow (4–7 Hz), middle (7–9 Hz), and fast (9–12 Hz) theta-range oscillations after formalin injection. The dashed lines and shaded areas indicate the mean and SEM at baseline, respectively. The noxious stimulus increased and decreased slow and fast theta power, respectively, during the nociceptive phases. (**b**) Scatter plots depicting nociceptive response plotted against theta power. Top: individual phases in individual animals. Bottom: values binned by time (2 min). There was a significant positive and negative correlation of nociceptive response with slow theta power (individual phases: R = 0.58, P = 0.0055; time-binned values: R = 0.45, P = 0.00000030) and fast theta power (individual phases: R = − 0.56, P = 0.0081; time-binned values: R = − 0.24, P = 0.0086), respectively. Individual phases: R = − 0.42, P = 0.061; time-binned values: R = 0.070, P = 0.44 for middle theta power. Data are presented as mean ± SEM; *P < 0.05 vs. baseline values; repeated measures ANOVA with a post hoc Dunnett’s multiple comparison test.

Thus far, our analyses indicated that theta-range oscillations were linked to a nociceptive response differentially across the frequency range. Next, we examined how theta oscillations in different frequency ranges correlated with the nociceptive response. Theta oscillations were subdivided into slow (4–7 Hz), middle (7–9 Hz), and fast (9–12 Hz) oscillations. We found that the power of slow theta oscillations increased during nociceptive phases but not during the interphase and at 24-h post-injection, although the difference did not reach statistical significance during phase 2 (Fig. 4a). Conversely, the fast theta power was significantly lower during phases 1 and 2 than that during the pre-injection phase, but not during the interphase and at 24-h post-injection (Fig. 4a). The middle theta power did not statistically differ between the pre- and post-injection phases (Fig. 4a).

We then tested any possible correlations between subdivided theta oscillations and nociceptive response. Scatter plots depicting nociceptive response plotted against each subdivided theta power are shown in Fig. 4b. A significant positive correlation was observed between a slow theta power and a nociceptive response, while a fast theta power was negatively correlated with a nociceptive response. This was the case for both a phase-by-phase basis and time-binned values. This relationship was not obtained between middle theta power and nociceptive response. Furthermore, nociceptive response did not show any significant change (Supplementary Figure S2 online) in or significant association (Supplementary Figure S2 online) with other frequency-range oscillations (i.e., delta, beta, and slow gamma oscillations). These data indicate that fast and slow theta powers are distinctively associated with pain intensity in the formalin-induced inflammatory pain model.

### Theta–gamma coupling during nociceptive response

**Figure 5:**
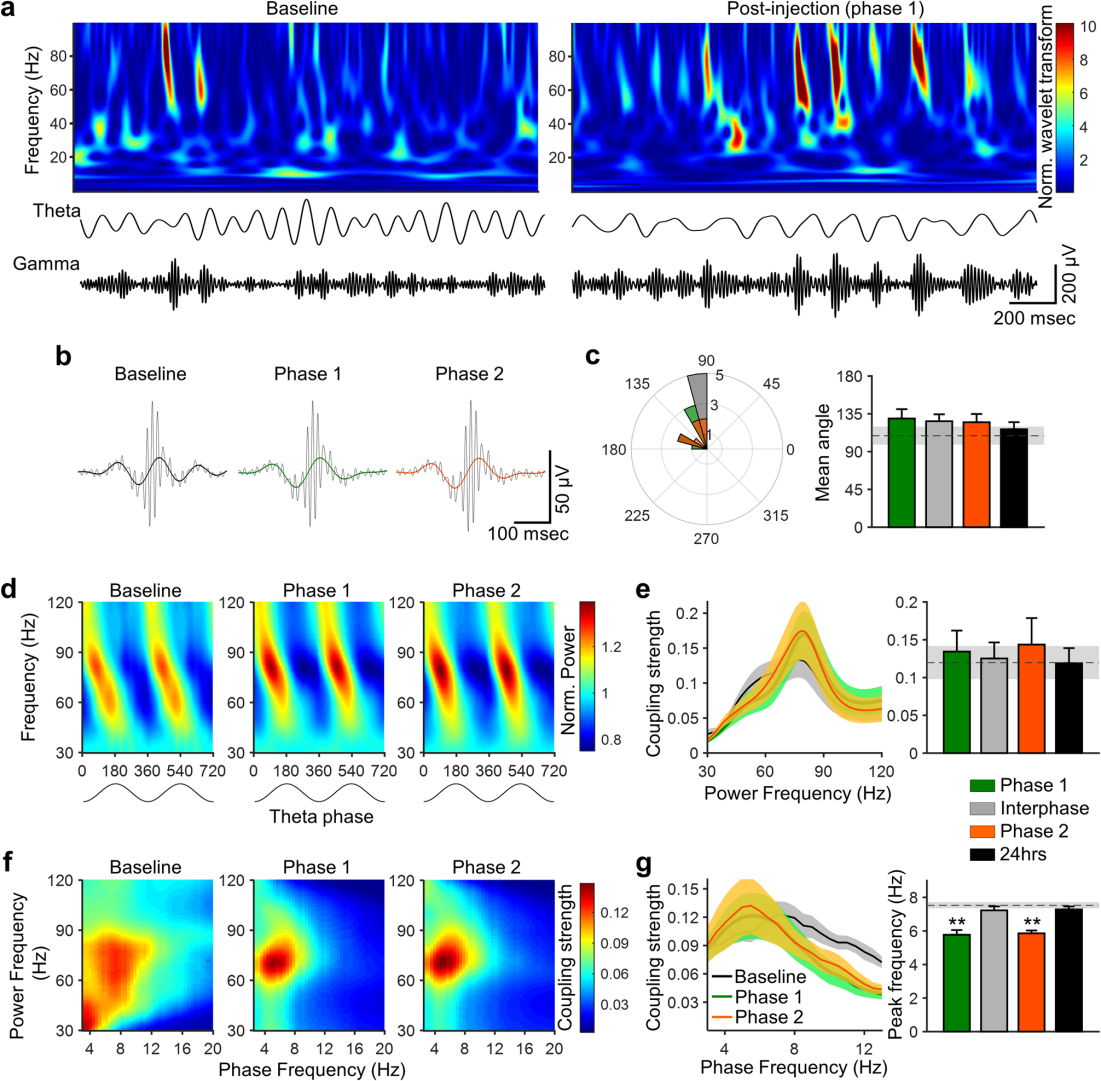
Cross-frequency coupling between theta phase and gamma power reveals peak shift of theta phase frequency during nociceptive phases. (**a**) Top: an example of normalized wavelet transform of local field potentials in the somatosensory cortex before (left) and after (right) formalin injection. Bottom: theta (4–12 Hz) and gamma (60–90 Hz) oscillations. (**b**) Averaged raw (black) and theta-range filtered (black: baseline, green: phase 1, orange: phase 2) traces obtained by aligning the signals at the peak of gamma oscillations. (**c**) Left: distribution of the mean direction of gamma power for the theta cycle phase. Right: mean direction of gamma power for the theta cycle phase in each phase. Mean direction did not differ after formalin injection. (**d**) Color-coded gamma power as a function of theta phase. (**e**) Left: strength of theta modulation of gamma as a function of power frequency. Right: averaged theta-gamma coupling. Theta-gamma coupling strength did not differ after formalin injection. (**f**) Phase-power comodulogram between power of high-frequency oscillation and phase of low-frequency oscillation at baseline and during nociceptive phases. (**g**) Left: coupling strength of theta modulation of gamma power as a function of phase frequency. Right: peak phase frequency of coupling strength, demonstrating peak frequency is significantly lower during phases 1 and 2, but not during the interphase or at 24-h post-injection. The dashed line and shaded area indicate the mean and SEM at baseline, respectively. Data are presented as mean ± SEM; *P < 0.05 vs. baseline values; repeated measures ANOVA with a post hoc Dunnett’s multiple comparison test.

Next, we examined if theta–gamma coupling changes were accompanied with a nociceptive response. LFPs have prominent theta and gamma oscillations that are revealed by wavelet transforms or bandpass filters (Fig. 5a, top and bottom, respectively)^17^. The gamma component, which was apparent within the range of 60–90 Hz, occurs most frequently at the peak and ascending phase of the theta oscillation, regardless of formalin injection (Fig. 5a). Indeed, the temporal dynamics of the cross-frequency coupling was left intact (Fig. 5b); the theta phase at which the gamma strength was the highest did not differ following formalin injection (Fig. 5c). In addition, the coupling strength of the theta phase and gamma power did not differ significantly because of the noxious stimulus (Fig. 5d,e). Furthermore, formalin injection did not affect the gamma power itself, regardless of the behavioral phase (Supplementary Figure S3 online). The distribution of instantaneous gamma frequency was also identical to baseline oscillations (Supplementary Figure S3 online). These data suggest that gamma-range oscillations are intact in the formalin-induced pain model.

Consistent with the peak shift of theta oscillations, the peak of coupling strength was shifted during the nociceptive phases. Figure 5f depicts comodulograms that represent the strength of coupling (warmer colors = stronger coupling) as a function of phase frequency (x-axis) and power frequency (y-axis). The coupling strength is shown in Fig. 5g as a function of phase frequency. These analyses revealed that the peak frequency of the theta phase was significantly slower during phases 1 and 2 than that at the baseline. This was not the case at the interphase and at 24-h post-injection (Fig. 5g).

This phenomenon was corroborated with a phase–phase coupling analysis. Phase–phase synchronization between two oscillators can be determined by correlating the instantaneous phase values of the oscillations^18^. We found that there was a correlation between the phase of theta oscillations and gamma oscillations, regardless of the formalin injection (Supplementary Figure S4 online). To quantitatively explore this coupling, we computed the mean resultant length (MRL) of the circular distribution from the difference between theta and gamma phases for different *n:m* relationships (Supplementary Figure S4 online). At baseline, this analysis yielded a prominent peak at approximately 9 *n:m* ratios, corresponding to nine possible gamma cycles within a theta period (Supplementary Figure S4 online). Meanwhile, the peaks during nociceptive phases were at approximately 11 Hz. This was significantly higher compared to that at baseline, indicating that more gamma cycles existed in a theta period during nociceptive phases than at baseline (Supplementary Figure S4 online), possibly reflecting slowing down of theta oscillations. In contrast, the peak MRL did not differ after formalin injection (Supplementary Figure S4 online). These data indicate that the theta-peak shift influences the temporal dynamics of cross-frequency coupling between theta and gamma oscillations without affecting the coupling strength.

## Discussion

In this study, we found that theta oscillations in the mouse S1 cortex were shifted toward a slower frequency in a formalin-induced acute inflammatory pain model. This change was observed when animals demonstrated pain-relevant behavior during phases 1 and 2, whereas the peak returned to normal during the interphase and at 24-h post-injection. Importantly, the theta-peak frequency was negatively correlated with pain intensity, indicating that the slowing down of theta oscillations was associated with the recognition of pain. We also found a significant inverse correlation between theta-peak frequency and height during pain perception. These values covaried during nociceptive phases, suggesting a link between the slowing down of theta frequency and the enhancement of theta peak. Finally, the peak of cross-frequency coupling between theta and gamma oscillations was also shifted toward a slow frequency without affecting the coupling strength of theta–gamma coupling. This finding was supported by an altered phase–phase relationship between theta and gamma oscillations.

The sensory thalamus can be a neural substrate underlying the pain-relevant changes of theta oscillations. Specifically, the ventral posterolateral nucleus (VPL) of the thalamus, which receives sensory input, is thought to be involved in pain perception^19^. Indeed, peripheral formalin injection alters the VPL neuronal firing pattern^20,21^, which is predominantly regulated by a GABAergic drive from the thalamic reticular nucleus (TRN)^22,23^. In a capsaicin-induced pain model, optical stimulation of TRN neurons increases the burst firing rate of VPL neurons and decreases the theta power in the S1 cortex associated with attenuated pain intensity^24^, suggesting that the TRN-VPL pathway in the thalamus is involved in the theta oscillation synchrony in the S1 cortex and processing of pain perception.

Dynamic changes in the firing pattern of thalamic neurons can relay sensory information to the cortex. Thalamic neurons fire in two dynamic and state dependent modes: a tonic mode and a burst mode^25^. These thalamic firing modes control pain information in the thalamus^26,27^. In a previously described mouse model of formalin-induced pain, the burst firing rate significantly decreased during phases 1 and 2^20^. Moreover, burst stimulation of VPL neurons alleviated formalin-induced pain. Additionally, VPL neuron burst firing reversed the pain-related S1 theta power activity and inhibited pain perception in a capsaicin-induced pain model^24^. These reports indicate that thalamic neuron burst firing can modulate the temporal pattern of theta oscillations in the S1 cortex during pain perception.

A recent report demonstrated that the peak of alpha oscillations generated in the human thalamus is lower than that generated in the cortex^28^. The authors determined the intracortical and thalamic generators of the alpha rhythm during quiet wakefulness^28^. Granger causal analysis revealed that the peak of alpha oscillations in the thalamo-cortical causality seemed to be lower than that in the cortico-thalamic causality^28^. These data support the idea that the pain-relevant theta-peak shift of the current findings resulted from an increased contribution from the thalamic input.

Pain stimuli from the peripheral nociceptors are conveyed to the cortex via multiple central ascending pathways. The lateral pain system, which involves the aforementioned lateral thalamus-S1 cortical pathway, mainly processes the sensory aspects of pain^29^. On the other hand, the medial pain system, which projects through the medial thalamus to the cortical regions including the anterior cingulate cortex and insular cortex, is responsible for the emotional aspects of pain^29^. Both systems have been reported to be disrupted in patients with chronic pain^30,31^. A previous study monitoring the brain activity in response to a pain stimulus in humans revealed that the insular cortex is the only cortical region showing a significant positive correlation between brain activation and pain ratings within subjects^32^. It has been reported that the insular cortex forms reciprocal connections with the S1 cortex^33,34^, and functional magnetic resonance imaging analysis in patients with chronic pain showed an augmented functional connectivity between the insular cortex and the S1 cortex^35^. These findings demonstrated that altered activity within the insular cortex or connectivity between the insular cortex and the S1 cortex can affect the dynamics of neural processing in the S1 cortex.

Theta-oscillation dynamics can be a clinical biomarker that quantitatively evaluates the ongoing status of pain sensation in humans. It is currently challenging to objectively predict pain intensity in patients with chronic pain that is preventing an appropriate pain treatment and clinical development of analgesic medicines^36^. The findings of the current study implicate that recordings of neural activity in the S1 cortex may be useful as an objective biomarker for pain. However, future studies are required to further investigate the translatability of these results from animal models to humans and to understand whether theta-oscillation in human patients with chronic pain are also dynamic, as we report in this study.

In conclusion, our findings highlight that theta oscillation dynamics in the S1 cortex represent ongoing pain sensations. The theta-peak shift was a reversible change, which was only observed during nociceptive phases, and inversely correlated with instantaneous pain intensity. These results suggest that the pain-related oscillation dynamics can be a predictive biomarker for ongoing status of pain sensation.

## Methods

### Animals and surgery

All animal experimental procedures were approved by the Institutional Animal Care and Use Committee of Mitsubishi Tanabe Pharma Corporation. All experiments were performed in accordance with the institutional and ARRIVE guidelines. Seven male C57BL/6J mice were purchased from Charles River Laboratories Japan (Kanagawa, Japan). Animals were housed in a temperature- and humidity-controlled room on a 12-h light/dark cycle and provided ad libitum access to water and standard commercial diet, CRF-1 (Oriental Yeast Co., Tokyo, Japan). Nine-week-old mice were anesthetized with isoflurane and placed in stereotaxic head holder. A single tungsten wire (75 µm) was implanted in the S1 cortex (− 0.5 mm anterior to bregma, 1.5 mm lateral to midline, 0.8 mm below brain surface). To verify the electrode position after recording, fluorescent dye (Vybrant DiI Cell-Labeling Solution, Invitrogen, Carlsbad, CA) was attached to the tip of the electrode. Skull screws were attached above the left olfactory bulb and the right cerebellum, which served as reference and ground, respectively. All wires were connected to a 36-channel interface board (EIB-36, Neuralynx, Tucson, AZ) anchored to a microdrive. Animals were allowed to recover from surgery for more than 5 days before the initiation of the following experiments.

### Formalin-induced pain model and electrophysiological recording

Mice were habituated in an arena (30 × 40 × 30 cm) for at least 10 min prior to the start of electrophysiological recordings with the TDT RZ5D base station (Tucker-Davis Technologies, Alachua, FL). LFP signals from the S1 cortex were recorded for 10 min before and 35 min following intraplantar injection of formalin (5%, 20 µL) into the left hind paw. Ten-minute duration recordings were also made for 24-h post-formalin injection to investigate the recovery from substance-induced pain. The duration of licking behavior of the formalin-injected hind paw was summed at 1-min intervals for a total duration of 35 min following formalin injection. Behavioral phases following formalin injection were defined as an initial phase of nociceptive response (phase 1, 0–10 min), a quiescent interphase (10–20 min), and a second phase of nociceptive response (phase 2, 20–35 min) based on the observed nociceptive response.

### Electrode verification

After the behavioral experiment was completed, animals were anesthetized with isoflurane and euthanized via exsanguination. Brains were removed and postfixed in 4% (w/v) paraformaldehyde dissolved in phosphate-buffered saline (PBS) at 4 °C for 24 h, followed by 30% (w/v) sucrose prepared in PBS for 3 days. Brains were embedded in OCT compound (Sakura Finetek, Tokyo, Japan) and sectioned on a cryostat (35 µm-thick). Fluorescent Nissl staining was performed using the NeuroTrace 500/525 green fluorescent Nissl stain (Invitrogen, Carlsbad, CA). Brain slices were incubated for 20 min with NeuroTrace (dilution 1:300) and washed with PBS. The sections were examined with a fluorescence microscope to verify electrode placement based on fluorescent dye placement during electrode insertion. All electrodes were verified to be in the appropriate locations (Supplementary Figure S1 online).

### Measurements

Data on LFP were imported into MATLAB (MathWorks, Natick, MA) for analysis. Custom written scripts and scripts provided by K. Harris (University College London), C. Torrence, and G. Compo (University of Colorado) were used.

#### Power analysis

The band power of delta, theta, beta, and gamma was defined as the average power in the frequency range of 1–4, 4–12, 12–30, and 60–90 Hz, respectively. To exclude the possibility that the behavioral state affects the LFP dynamics after formalin injection, LFPs were used for analysis only when the animals were in a non-moving state (i.e., locomotor speed ≤ 4 cm/s). The power of LFPs was computed with the ‘‘pwelch’’ function in MATLAB. Locomotor speed was computed with the position of the animal in the arena monitored using two light-emitting diodes mounted on the headstage.

#### Instantaneous analysis

Field potentials in the theta range (4–12 Hz) were filtered using a zero-phase-delay filter as previously described^17^. The filter uses a linear phase function (fir1; MATLAB Signal Processing Toolbox, MathWorks) and compensates for the phase delay by time-shifting the filtered signal. Instantaneous frequency was computed from the instantaneous derivative of the phase time series.

#### Phase-power coupling

To examine the hierarchical relationship between theta frequency-range and gamma frequency-range oscillations, theta–gamma coupling was computed as previously described^17^. Briefly, the Morlet wavelet transform (1–150 Hz, a length of two cycles) was calculated with the wavelet software package (http://paos.colorado.edu/research/wavelets/software.html) to analyze LFP changes in multiple frequency domains. LFP power in each frequency over time was represented by the square of absolute values of the result of wavelet convolutions. To calculate the phase of ongoing theta oscillations, LFP signals were filtered in the theta range with a zero-phase-delay filter. The phase of the filtered LFP was then computed using the Hilbert transform. To measure the strength of theta–gamma coupling, theta phases were binned into *π*/50 intervals (0–360°) and the mean power of the gamma waves in each phase bin was calculated. The resulting values were input as weights to calculate the MRL, which takes a value between 0 (no coupling) and 1 (perfect coupling). To calculate the comodulogram between low-frequency phase and high-frequency power, bands of low-frequency activity were extracted with a bandpass width of 0.5 Hz (centers at 4–20 Hz), and phase-power coupling was measured for wavelet power between 30–150 Hz.

#### Gamma peak analysis

Gamma peak analysis was performed as previously described^17^. LFP signals were filtered at the gamma frequency range. Then, a time series indicating the peak times of the filtered signal was constructed, with the requirement that peak times were separated by at least 100 ms from each other (i.e., just the highest peaks within 100-ms windows were used to avoid selecting multiple peaks within a theta cycle). Peak-averaged theta waves were obtained by averaging 200-ms epochs of theta signals centered at the time points corresponding to gamma peaks. The mean phase of theta waves was computed by averaging the theta phases in which the gamma peak existed.

#### Phase–phase coupling

Phase–phase coupling was analyzed as previously described^18^. Phase coupling between two oscillators occurs in an *n*:*m* ratio, when there are *m* cycles of the “driven” oscillator for every *n* “stimulus,” which means that if there is a consistent *n*:*m* relationship, the difference between n*theta phase and m*gamma phase should have a consistent value. *n*:*m* coupling patterns were analyzed between theta-range frequency and gamma-range frequency oscillations. The MRL of the distribution of the difference between n*theta phase and m*gamma phase was calculated for variable *n*:*m* ratios as shown in Supplementary Figure S4 online.

### Statistical analysis

Data are represented as mean ± SEM. All statistical testing was conducted in MATLAB using the Statistics and Machine Learning Toolbox. Measures of central tendency were used to summarize band power at different frequencies. To correct for multiple comparisons for testing the phase-dependent effect of formalin injection on oscillatory dynamics, repeated measures analysis of variance with the post-hoc Dunnett’s multiple comparison test was used (Figs. 1c,f, 4a, 5e,g, Supplementary Figures S2, S3, and S4 online). Student’s *t*-test with Bonferroni correction was used for examining the difference of instantaneous waves among behavioral phases (Fig. 3c,d, and Supplementary Figure S3 online). The Watson–Williams test was used for examining the equality of the mean direction of the theta–gamma coupling (Fig. 5c). Pearson’s correlation coefficient test was used to assess for the correlations between the theta-peak frequency and height or the nociceptive response and metrics of oscillatory dynamics (Figs. 2, 3a, 4b, and Supplementary Figure S2 online).

## Supplementary Information

Supplementary Information

## Data Availability

The datasets generated during and/or analyzed during current study and the custom written scripts used in this study are available from the corresponding author on reasonable request.
