# Supplementary material for: Dynamics of neuronal oscillations underlying nociceptive response in the mouse primary somatosensory cortex
Source: Sci Rep. 2021 Jan 18;11:1667. doi: 10.1038/s41598-021-81067-0 (PMC7813887; doi:10.1038/s41598-021-81067-0)
Supplement: Supplementary file 1 — Supplementary Information [file 41598_2021_81067_MOESM1_ESM.docx]

**Dynamics of neuronal oscillations underlying nociceptive response in the mouse primary somatosensory cortex**

Shosuke Iwamoto^ǂ^, Makoto Tamura*^ǂ^, Atsushi Sasaki, and Masao Nawano

Neuroscience Research Unit, Mitsubishi Tanabe Pharma Corporation, Yokohama, Kanagawa 227-0033, Japan

ǂ These authors contributed equally to the work.

* Corresponding author

Makoto Tamura Ph.D.

Neuroscience Research Unit, Mitsubishi Tanabe Pharma Corporation

1000, Kamoshida-cho, Aoba-ku, Yokohama Kanagawa, 227-0033, Japan

Tel: +81-45-963-4681

Fax: +81-45-963-4641

Email: tamura.makoto@mu.mt-pharma.co.jp

**Supplementary Materials**

**Supplementary Figure S1.** Electrode placement verification. (**a**) Representative images of fluorescent Nissl-stained brain sections; the arrow indicates electrode tip location. (**b**) Schematic coronal sections of the mouse brain atlas^37^ showing electrode placements in the primary somatosensory (S1) cortex; numbers indicate distance anterior to bregma.

**Supplementary Figure S2.** Delta, beta, and slow gamma power after formalin injection. (**a**) Mean power in delta- (1–4 Hz), beta- (12–30 Hz), and slow gamma- (30–60 Hz) range oscillations following formalin injection. The dashed line and shaded area indicate the mean and SEM at baseline, respectively. The noxious stimulus did not affect the power of these oscillations. (**b**) Scatter plots depicting the nociceptive response plotted against power. Top: Individual phases in individual animals; bottom: values binned by time (2 min). No significant correlation of nociceptive response with power was observed, regardless of frequency (Delta, individual phases: R = 0.36, p = 0.11; time-binned values: R = 0.080, p = 0.39; Beta, individual phases: R = 0.22, p = 0.34; time-binned values: R = 0.18, p = 0.051; slow gamma, individual phases: R = 0.15, p = 0.53; time-binned values: R = 0.12, p = 0.19). Data are presented as mean ± SEM.

**Supplementary Figure S3.** Gamma power after formalin injection. (**a**) Power spectra in the somatosensory cortex at baseline and during nociceptive phases. (**b**) Mean gamma power did not significantly differ following formalin injection. The dashed line and shaded area indicate the mean and SEM at baseline, respectively. (**c**) The probability of the instant frequency of gamma was not different following formalin injection.

**Supplementary Figure S4.** Phase-phase coupling between theta and gamma oscillations. (**a**) Phase-phase plots at baseline and during nociceptive phases. (**b**) Examples of the distribution of the difference between theta and gamma phases for two different n:m relationships (blue, 1:9; yellow, 1:11) at baseline and during phase 1. (**c**) Mean resultant length (MRL) values from the distribution of phase differences, calculated for n*theta–m*gamma phase, as a function of the n:m ratio at baseline (black), during phase 1 (green) and phase 2 (orange). (**d** and **e**) Mean peak n:m ratio (**d**) and peak MRL (**e**) following formalin injection. The dashed lines indicate the mean values at baseline. Peak n:m ratio was significantly higher during phases 1 and 2 than at baseline, but not during the interphase and at 24-h post-injection (**d**). Peak MRL did not differ after formalin injection, regardless of the behavioral phase (**e**). Data are presented as mean ± SEM. * P < 0.05, ** P < 0.01 vs. baseline values; repeated measures ANOVA with a post hoc Dunnett’s multiple comparison test.

**Supplementary Figure S1**
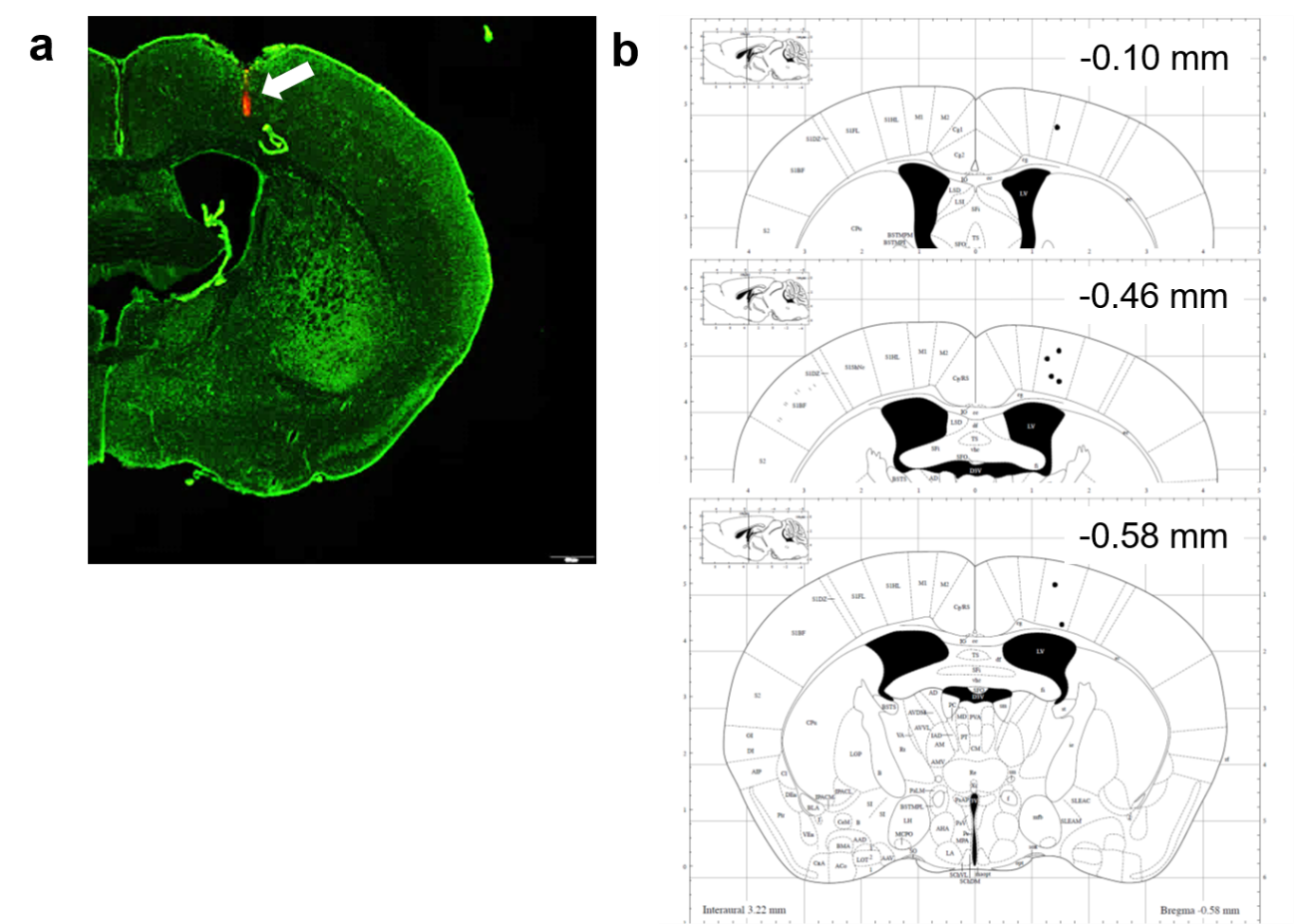


**
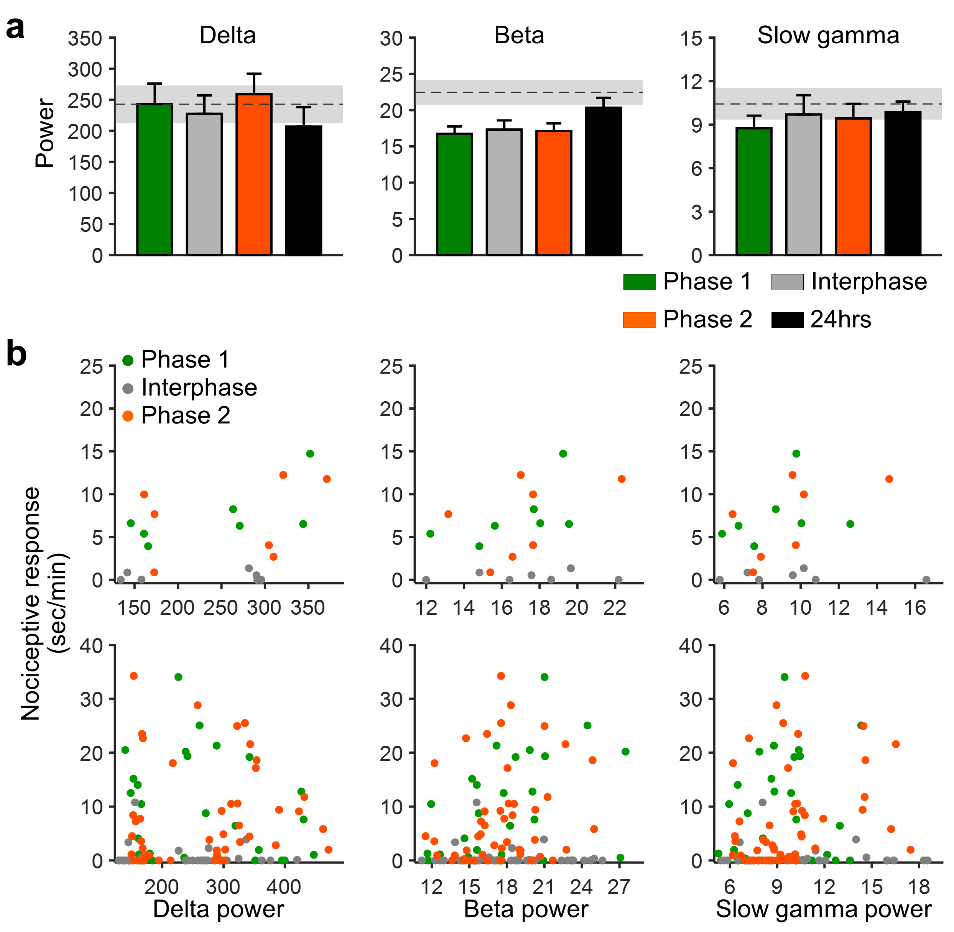
Supplementary Figure S2**

**
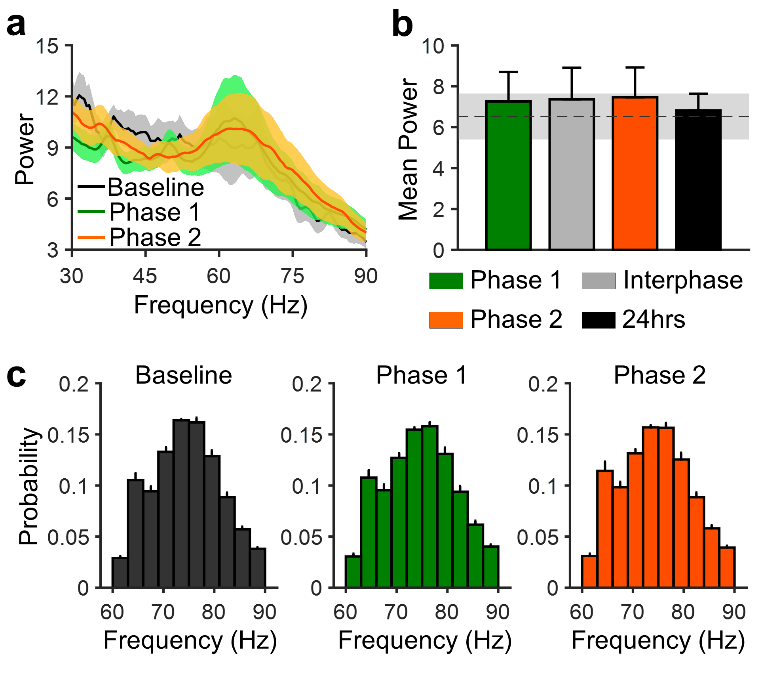
Supplementary Figure S3**


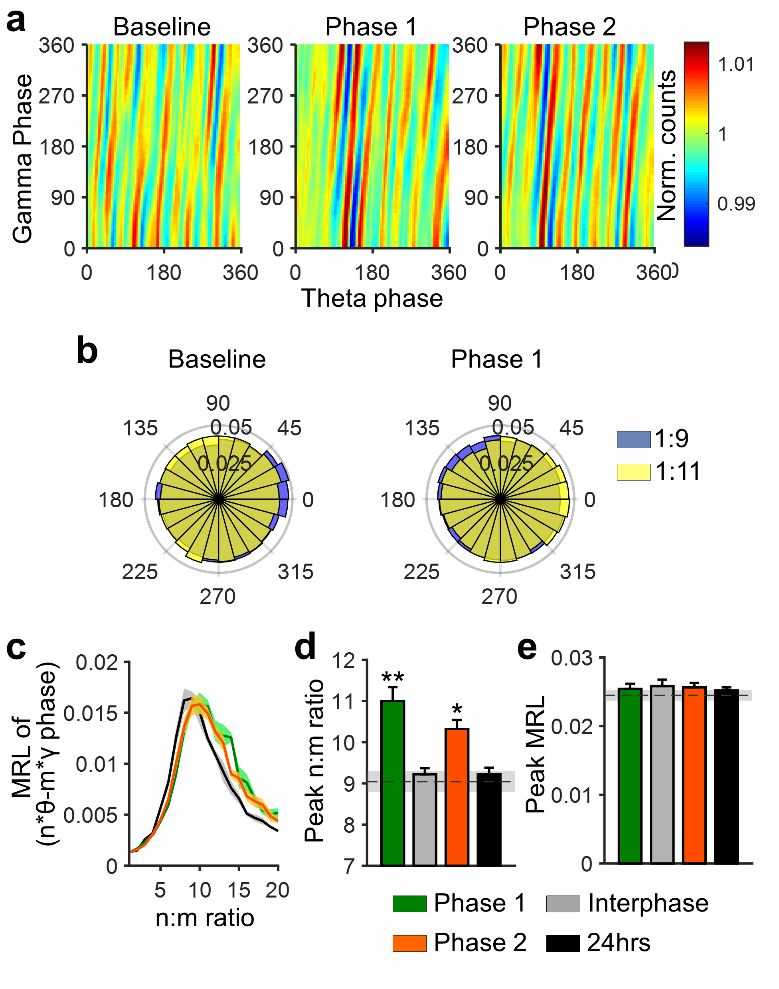
**Supplementary Figure S4**

**Reference**

37 Paxinos, G. & Watson, C. *The rat brain in stereotaxic coordinates Ed 4. San Diego, CA: Academic Press*. (1998).
